# Data on a cytoarchitectonic brain atlas: effects of brain template and a comparison to a multimodal atlas

**DOI:** 10.1016/j.dib.2017.04.007

**Published:** 2017-04-13

**Authors:** Mona Rosenke, Kevin S. Weiner, Michael A. Barnett, Karl Zilles, Katrin Amunts, Rainer Goebel, Kalanit Grill-Spector

**Affiliations:** a Department of Psychology, Stanford University, Stanford, CA, USA; b Institute for Neuroscience and Medicine (INM-1), and JARA Brain, Research Centre Jülich, Jülich, Germany; c Department for Psychiatry, Psychotherapy and Psychosomatics, University Hospital Aachen, RWTH Aachen University, and JARA-BRAIN, Aachen, Germany; d C. and O. Vogt Institute for Brain Research, Heinrich Heine University Düsseldorf, Germany; e Faculty of Psychology and Neuroscience, Maastricht University, The Netherlands; f Netherlands Institute for Neuroscience, Amsterdam, The Netherlands; g Stanford Neurosciences Institute, Stanford, CA, USA

## Abstract

The data presented here are related to the research article: “*A cross-validated cytoarchitectonic atlas of the human ventral visual stream”* in which we developed a cytoarchitectonic atlas of ventral visual cortex. Here, we provide two additional quantifications of this cytoarchitectonic atlas: First, we quantify the effect of brain template on cross-validation performance. The data show a comparison between cortex-based alignment to two templates: the postmortem average brain and the FreeSurfer average brain. Second, we quantify the relationship between this cytoarchitectonic atlas and a recently published multimodal atlas of the human brain (Glasser et al., 2016).

**Specifications Table**

**Table:** 

Subject area	*Neuroscience*
More specific subject area	*Brain mapping, cytoarchitecture, cortical surface templates, atlas comparison*
Type of data	*Figures; maximum probability cytoarchitectonic atlas on the FreeSurfer average brain*
How data was acquired	*MRI; postmortem brain slices that were cell-body stained using the Merker method*[9]
Data format	*Analyzed*
Experimental factors	*Single subject analyses; group analyses after alignment to a common brain space*
Experimental features	*(1) Quantifying the effect of the cortical surface template used for alignment on cross-validation performance of a cytoarchitectonic atlas in occipital and ventral temporal cortex.*
	*(2) Quantifying the correspondence between this cytoarchitectonic atlas and a multimodal brain atlas.*
Data source location	*Germany; USA*
Data accessibility	*Data are published on*http://vpnl.stanford.edu/vcAtlas*and are related to the research article*[10].
Related research article	*Rosenke, M., Weiner, K.S., Barnett, M.A., Zilles, K., Amunts, K., Goebel, R., Grill-Spector, K. A cross-validated cytoarchitectonic atlas of the human ventral visual stream.*

**Value of the data**

• The data show the effect of cortical surface template on the precision of a cytoarchitectonic brain atlas.
• Comparison between the cytoarchitectonic atlas and a multimodal atlas will allow researchers to investigate commonalities as well as deviations between these two recent parcellations of the human brain.

## 1 Data

The data are based on Magnetic Resonance Images (MRI) and histological analyses of human postmortem brains. Histological slices of each brain were co-registered to the respective whole brain MRI scan. Data were acquired in 11 postmortem (PM) brains. Using these data, 8 cytoarchitectonic regions of interest (cROIs) where identified. 4 regions were defined in the occipital lobe: human Occipital cortex 1 (hOc1) and hOc2 [1], hOc3 ventral (hOc3v) and hOc4v [11], Fusiform Gyrus 1 (FG1) and FG2 [2], and FG3 and FG4 [8]. Details about original area definitions can be found in the respective publications.

All 8 cytoarchitectonic areas were identified in 9 brains; in the 10th brain, all of the occipital cROIs and two of the FG cROIs (FG1 and FG2) were identified, but not FG3 and FG4 because histological processing resulted in distortions in the regions of interest. Since we planned to include data from 10 brains for the calculation of probability maps for each cytoarchitectonic area in the main research article, we added an 11th brain cROIs FG3 and FG4 were identified.

## 2 Experimental design, materials and methods

Each of the MRIs of the postmortem brain anatomies was manually segmented to separate gray from white matter using ITK-SNAP (http://www.itksnap.org/pmwiki/pmwiki.php). The segmentations were then used to create a cortical surface reconstruction for each individual brain and each hemisphere, separately. Subsequently, each brain׳s anatomical T1-weighted image, cortical segmentation, and cytoarchitectonic areas were further analyzed in BrainVoyager QX 2.8 (Brain Innovation, Maastricht, The Netherlands) and FreeSurfer (http://surfer.nmr.mgh.harvard.edu). Cytoarchitectonic areas were projected from each brain׳s volume to their cortical surface reconstruction. We then used cortex-based alignment [4], [7] to register each brain and the respective cROIs to a common brain template in which we generated a group probability map of each cROI. Details about atlas generation are described in Rosenke et al. [10].

### 2.1 Examining the effect of cortical surface template on cross-validated atlas performance

Here, we examined how the choice of brain template used for the alignment affects the precision of the atlas. We compared two templates: a postmortem brain average created with our PM brains and the FreeSurfer average brain (fsaverage, [4]). In this comparison, the cROI atlas was generated using data from 9 brains in which all 8 cROIs of the human ventral visual stream were identified.

#### 2.1.1 Cortex-based alignment to the postmortem group average surface (CBApm)

Each brain׳s left and right cortical surface was inflated into a sphere and curvature maps of gyri and sulci were created. Then, each brain was rotated to best match the curvature pattern of a randomly chosen target brain using an initial rigid alignment. The best match was established by the lowest variability in curvature between each respective brain and the target brain (Details in [5], [7]). Second, a non-rigid cortex-based alignment was initiated by iteratively aligning curvature maps of all PM brains to each other through vertex movements. This process is done iteratively, yielding a coarse to fine curvature alignment with four levels of anatomical detail. During this process, a continuously updated group average curvature map is created. The endproduct of this iterative process is an average curvature map of the 9 postmortem brains (PM9), which was then used to create the PM9 average cortical surface. During this alignment, each of the 9 individual anatomies contribute an equal weight to the formation of the PM9 cortical surface. For each PM brain, a sphere-to-sphere mapping file, which aligns the individual brain׳s cortical surface to the PM9 cortical surface, is created. This mapping is then applied to the individual brain׳s cytoarchitectonic areas, bringing them into alignment to the common PM9 cortical surface.

#### 2.1.2 Cortex-based alignment to the FreeSurfer atlas (CBAfs)

This transformation is similar to the alignment to the postmortem group average (CBApm), except that each brain׳s left and right cortical surface was aligned to the FreeSurfer average brain (fsaverage), for both the rigid and non-rigid alignment. The fsaverage cortical surface reflects the average cortical surface of 39 independent living adults and is therefore independent of the postmortem data. For each postmortem hemisphere, the sphere-to-sphere mapping file aligning the individual brain׳s cortical surface to the fsaverage was saved during the alignment process. This transformation was applied to each cytoarchitectonic areas to bring them into alignment with the fsaverage cortical surface.

#### 2.1.3 Evaluating the cross-validation performance of the cROI atlas across the two templates

We quantified how well a group cROI predicts cROIs of individual brains using an exhaustive leave-one-out cross-validation procedure. In each iteration, we created a group probabilistic map of each cROI based on all brains but one, and tested how well it predicts the location and extent of the cROI in the left-out brain. This procedure was repeated for all combinations of left-out brains, and separately for each alignment methods. We estimated the predictability of the group probabilistic cROI (*G*) and the left-out cROI (*I*) by calculating the dice coefficient (*dc*) between these cROIs:

$$\mathrm{dc}=\frac{2|I\cap G|}{\left|I\right|+|G|}$$

**Fig. 1:**
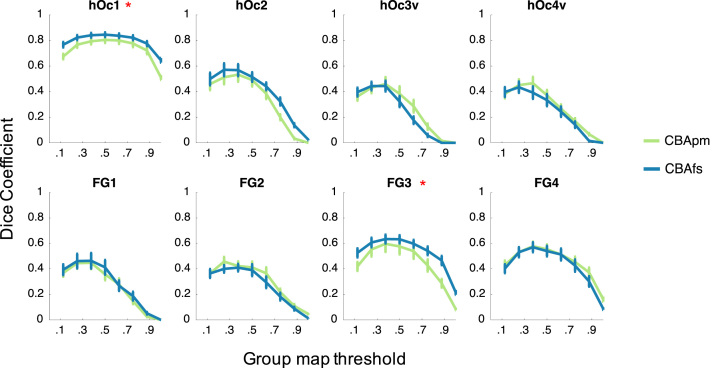
Leave-one-out cross-validation performance of CBApm and CBAfs. Each panel shows the cross-validated dice coefficient for each cROI as a function of the threshold applied to the group cROI map. The threshold determines the minimal proportion of overlapping brains at the vertex level included in the group cROI. *Green (CBApm):* cortex-based alignment to the PM9 cortical surface. *Cyan (CBAfs):* cortex-based alignment to the fsaverage cortical surface. *Error bars:* standard error (SE) of performance across the 9-fold cross-validation. *Asterisks:* significant difference (*P*<0.05) between the dice coefficient of CBApm and CBAfs measured at two thresholds: no threshold and .33.

The dice coefficient is a statistic used for comparing the similarity of two samples 12], [3]. A dice coefficient of zero indicates no predictability and a dice coefficient of 1 indicates perfect predictability. We applied different threshold levels to the group probabilistic cROI (G) to predict the location of the left-out-brain (Fig. 1). Thresholds ranged from an unthresholded group probabilistic map to a very conservative threshold where only vertices present in all subjects were included in the group map. Thresholds increased in steps of. 125, which is equivalent to one subject out of the total 8 used to generate the group cROI. Alignments were statistically evaluated at a threshold level of .375 (including vertices shared by more than two subjects), as well as with unthresholded data. The threshold of two or more subjects per vertex was found to have the highest predictability across cROIs and different alignment methods (see [10]). Differences between the predictability of the two surface templates were evaluated using permutation testing with replacement using 10,000 permutations in which cROI alignments were randomly shuffled. Significant differences (*P*​<.05) in prediction performance across the two alignments is indicated by asterisks in Fig. 1.

### 2.2 Comparison between the cytoarchitectonic atlas and a multimodal atlas of the human brain

The final cytoarchitectonic atlas was generated on the fsaverage (using CBAfs) containing data from 10 brains per cROI. The atlas is available in both BrainVoyager and FreerSurfer formats and can be downloaded here: http://vpnl.stanford.edu/vcAtlas.

**Fig. 2:**
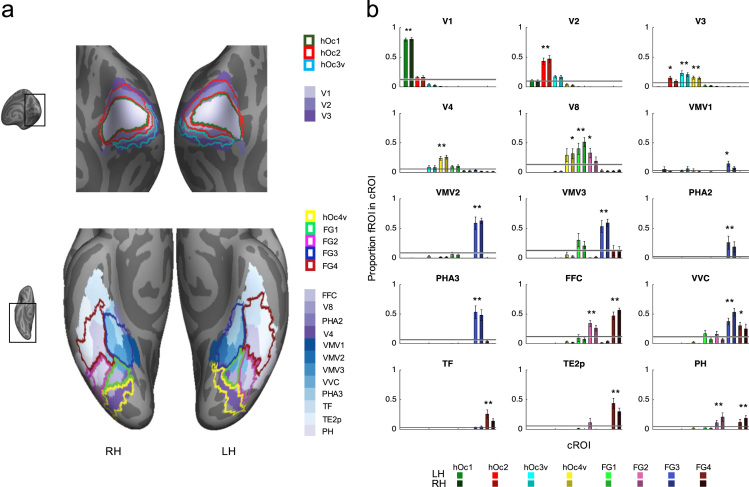
Coupling between cytoarchitectonic and multimodal atlases of the human ventral visual stream. Data are compared between our cROI atlas and the multimodal atlas published by Glasser et al. [6]. (a) Superposition of the cROI atlas (outlines) and the Glasser atlas (solid) on the fsaverage brain. *Top:* occipital view of the fsaverage brain, inset shows zoomed region of the brain displayed. *Bottom:* ventral view of the fsaverage brain; *Solid colors:* multimodal ROIs (mROIs); *Outlines:* cROIs. (b) Quantification of correspondence between mROIs and cROIs. Each graph shows the average overlap between a mROI and each individual cROI projected to the fsaverage brain. Data are shown for left hemisphere (light colors) and right hemisphere (dark colors) separately. *X-axis:* cROIs. *Y-axis:* proportion of mROI contained in each cROI. *Horizontal bars:* chance level with 95% confidence interval. *Errorbars:* standard error across PM brains. *Asterisks*: Significantly different from chance level (*P*<0.05).

These cytoarchitectonic areas can be compared to other parcellations of the ventral visual stream such as those derived from other anatomical or functional metrics. To demonstrate this, we compared the present cytoarchitectonic atlas to a recently published multimodal atlas by Glasser et al. [6]. We calculated the proportion of overlap between each multimodal region (mROI) in ventral occipital or ventral temporal cortex and each of the cytoarchitectonic areas on the fsaverage brain (Fig. 2b). The overlap was calculated between the mROIs and each of the individual PM׳s cROIs. Data shown in Fig. 2 show the average overlap across 10 PM brains. We performed this analysis for all mROIs published by Glasser and colleagues [6] that were in the same cortical expanse of the cytoarchitectonic areas included in this ventral cytoarchitectonic atlas. This includes the following mROIs: V1, V2, V3, V4, V8, FFC, PHA2, PHA3, VMV1, VMV2, VMV3, VVC, TF, TE2p and PH. Additionally, we computed an mROI-specific chance level to test if the overlap between a given mROI and cROI is significantly different from chance. We estimated the chance level by calculating the average percentage overlap between a given mROI and a randomly chosen cROI (of the 8 cROIs) from a randomly chosen brain (of the 10 PM brains). This procedure was repeated 1000 times with replacement. Then we tested if the measured overlap was significantly greater than chance (*P*<0.05, Bonferroni corrected for multiple comparison of cROIs (8) with each mROI) for each hemisphere separately. Both hemispheres were evaluated separately as we found a main effect for hemisphere (Friedman׳s test: chi-sq(1,119) = 4.38, *P*<.05).

Comparison between the atlases is shown in Fig. 2. Greater than chance coupling between mROIs and cROIs is indicated by asterisks in Fig. 2.

## References

[1] Amunts K., Malikovic A., Mohlberg H., Schormann T., Zilles K. (2000). Brodmann׳s areas 17 and 18 brought into stereotaxic pace - where and how variable?. Neuroimage.

[2] Caspers J., Zilles K., Eickhoff S.B., Schleicher A., Mohlberg H., Amunts K. (2013). Cytoarchitectonical analysis and probabilistic mapping of two extrastriate areas of the human posterior fusiform gyrus. Brain Struct. Funct..

[3] L.R. Dice, Measures of the Amount of Ecologic Association Between Species Author ( s): Lee R. Dice Published by: Wiley Stable URL: 〈http://www.jstor.org/stable/1932409〉 Accessed: 08-04-2016 13: 33 UTC Your use of the JSTOR archive indicates your acceptance of th. Ecology 26, 297–302, 1945.

[4] Fischl B., Sereno M.I., Tootell R.B., Dale A.M. (1999). High-resolution intersubject averaging and a coordinate system for the cortical surface. Hum. Brain Mapp..

[5] Frost M.A., Goebel R. (2012). Measuring structural-functional correspondence: spatial variability of specialised brain regions after macro-anatomical alignment. Neuroimage.

[6] Glasser M.F., Coalson T.S., Robinson E.C., Hacker C.D., Harwell J., Yacoub E. (2016). A multi-modal parcellation of human cerebral cortex. Nat. Publ. Gr..

[7] Goebel R., Esposito F., Formisano E. (2006). Analysis of functional image analysis contest (FIAC) data with brainvoyager QX: from single-subject to cortically aligned group general linear model analysis and self-organizing group independent component analysis. Hum. Brain Mapp..

[8] Lorenz S., Weiner K.S., Caspers J., Mohlberg H., Schleicher A., Bludau S., Eickhoff S.B., Grill-Spector K., Zilles K., Amunts K. (2015). Two new cytoarchitectonic areas on the human mid-fusiform gyrus. Cereb. Cortex bhv225.

[9] Merker, B., 1983. Silver staining of cell bodies by means of physical development. J. Neurosci. Methods 9, 235–241. 10.1016/0165-0270(83)90086-9 6198563

[10] Rosenke M., Weiner K.S., Barnett M., Zilles K., Amunts K., Goebel R., Grill-Spector K. (2017). A cross-validated cytoarchitectonic atlas of the human ventral visual stream. Neuroimage..

[11] Rottschy C., Eickhoff S.B., Schleicher A., Mohlberg H., Kujovic M., Zilles K., Amunts K. (2007). Ventral visual cortex in humans: cytoarchitectonic mapping of two extrastriate areas. Hum. Brain Mapp..

[bib12] Sørensen T. (1948). A method of establishing groups of equal amplitude in plant sociology based on similarity of species and its application to analyses of the vegetation on Danish commons. Biol. Skr..

